# Gut Microbiota in Lipodystrophies and Obesity: A Common Signature?

**DOI:** 10.3390/microorganisms14010132

**Published:** 2026-01-07

**Authors:** Luca Colangeli, Adelaide Teofani, Alessandro Desideri, Silvia Biocca, Teresa Pacifico, Maria Eugenia Parrotta, Veronica Fertitta, Paola Fortini, Giovanni Ceccarini, Silvia Magno, Caterina Pelosini, Ferruccio Santini, Giuseppe Novelli, Paolo Sbraccia, Valeria Guglielmi

**Affiliations:** 1 Obesity Medical Center, University Hospital Policlinico Tor Vergata, Viale Oxford 81, 00133 Rome, Italy; 2 Department of Systems Medicine, University of Rome Tor Vergata, Via Montpellier 1, 00133 Rome, Italy; 3 Department of Biology, University of Rome Tor Vergata, Via della Ricerca Scientifica 1, 00133 Rome, Italy; 4 Department of Environment and Health, Istituto Superiore di Sanità, Viale Regina Elena 299, 00161 Rome, Italy; 5 Obesity and Lipodystrophy Center, Endocrinology Unit, University Hospital of Pisa, Via Paradisa 2, 56124 Pisa, Italy; 6 Department of Biomedicine and Prevention, Medical School, University of Rome Tor Vergata, Via Montpellier 1, 00133 Rome, Italy

**Keywords:** lipodystrophy, obesity, gut microbiota, metabolically healthy obesity, metabolically unhealthy obesity, dysbiosis, adiposopathy

## Abstract

Lipodystrophies are rare syndromes characterized by partial or complete loss of subcutaneous adipose tissue leading to ectopic lipid deposition, insulin resistance, and the same metabolic derangements observed in obesity. Given the role of gut microbiota in metabolic disorders, we investigated whether its signature in obesity may be mirrored by that found in lipodystrophies, possibly contributing to their overlapping metabolic abnormalities. In this cross-sectional study, we included 8 individuals with lipodystrophy (LD), 16 individuals with obesity (Ob)—further categorized into 8 metabolically healthy (MHO) and 8 metabolically unhealthy (MUHO)—and 16 normal-weight controls (N). We assessed clinical and metabolic characteristics and performed 16S rRNA sequencing and bioinformatic analyses on fecal samples to characterize the gut microbiome. LD presented significantly lower body mass index (BMI) and waist circumference than Ob, but, from a metabolic perspective, LD showed similarity with MUHO and presented significantly lower levels of HDL-C and higher triglycerides compared to both N and MHO. Gut microbiota analysis revealed reduced α-diversity in LD, MHO and MUHO compared to N, whilst β-diversity and Firmicutes/Bacteroidetes ratio differences were not significant. At the phylum level, differential abundance analysis revealed that LD individuals exhibit similar microbial characteristics to MUHO and higher *Verrucomicrobiota* levels compared to MHO. The shared gut microbiota signature suggests another potential unexplored link between the pathogenesis of metabolic complications in lipodystrophies and obesity, providing novel insights into the complex interplay between dysbiosis and adiposopathy. Larger longitudinal studies are needed to explore the role of specific taxa and for a more precise characterization of different lipodystrophy subtypes.

## 1. Introduction

Lipodystrophies (LD) are a heterogeneous group of congenital or acquired rare syndromes characterized by a partial or generalized loss of subcutaneous adipose tissue [1,2,3]. The loss of subcutaneous fat in lipodystrophies mirrors the ‘adiposopathy’ and limited expandability of the same depot in obesity, turning into ectopic lipid deposition, lipotoxicity and insulin resistance development [4]. As a consequence, despite the opposite phenotype, obesity and lipodystrophies converge on the same cardiometabolic complications such as type 2 diabetes, dyslipidemia, hepatic steatosis and cardiovascular diseases [5].

Based on the metabolic status, individuals with obesity can be further categorized into individuals with metabolically unhealthy obesity (MUHO) and individuals with metabolically healthy obesity (MHO) [6], even though the latter mostly represents a transient phenotype deemed to develop cardiometabolic complications over time [7]. With regard to adiposopathy, in contrast to MHO, in individuals with MUHO adipose tissue remodeling is maladaptive, entailing adipocyte hypertrophy, disrupted vascularization, heightened pro-inflammatory microenvironment and a more evident accumulation of lipids at ectopic sites [8].

In recent years, gut microbiota has been identified as a key player in the development and progression of metabolic diseases [9] through mechanisms that remain incompletely understood, including dysregulated inflammatory pathways [10] and disruption of the gut–brain axis. Alterations in microbial composition can promote low-grade systemic inflammation, modify neuroimmune communication, and ultimately impair the central regulation of appetite and energy homeostasis, thereby contributing to metabolic dysfunction [11]. Restoring a healthy microbial community through the use of probiotics, prebiotics or fecal microbiota transplantation can help rebalance the gut ecosystem and attenuate inflammation both locally and systemically, leading to improvements in both gastrointestinal and systemic conditions [12]. Indeed, evidence suggests that greater richness and diversity in gut microbiota is associated with better nutritional status, fewer comorbidities and overall improved health, while individuals with metabolic impairments generally present low diversity [9]. Patients affected by obesity have been reported to have a reduction in Firmicutes/Bacteroidetes ratio [13] as compared to normal weight controls. When applying to MHO and MUHO categories, the latter commonly presents a gut microbiota with a more inflammatory profile, and lower diversity and Firmicutes/Bacteroidetes ratio [14,15,16], suggesting that gut microbiota changes may contribute to the development of obesity complications. Regarding LD, to the best of our knowledge, only a single study examined the gut microbiota composition in such patients [17], reporting an altered microbial profile.

In this context, the aim of our study was to analyze the gut microbiota of patients with LD in comparison with individuals with obesity, to determine whether they display comparable patterns of dysbiosis. By conducting the study, we sought to expand current knowledge on this rare disease while providing insights into the mechanisms underlying metabolic impairment in adiposopathy.

## 2. Materials and Methods

### 2.1. Study Population

In this cross-sectional study, we recruited 8 patients with lipodystrophy (LD), 16 patients with obesity (Ob), defined as BMI ≥ 30 kg/m^2^, and 16 normal-weight controls (N). Ob participants were further classified into metabolically healthy obesity (MHO; *n* = 8) and metabolically unhealthy obesity (MUHO; *n* = 8), according to their metabolic status [18].

Inclusion criteria included Caucasian origin and stable body weight for at least 3 months preceding the study. Exclusion criteria were chronic liver or kidney disease, active infection, malignancy, other acute or chronic systemic diseases, recent hospitalization (<30 days), use of glucocorticoids, nonsteroidal anti-inflammatory drugs, antibiotics, prebiotics, and probiotics within 3 months before evaluation, and pregnancy or breastfeeding.

The enrolled participants underwent a detailed medical history assessment, and anthropometric (body weight, height, and waist circumference) and clinical (blood pressure and heart rate) evaluation. Biological (blood and stool) samples were collected.

The study was approved by the Ethical Committee of the University Hospital “Policlinico Tor Vergata” and all patients gave written informed consent.

### 2.2. Biological Samples

Blood samples were collected in the morning from fasting participants and analyzed immediately or processed and stored in aliquots at −80 °C (whole blood, serum and plasma) until use.

Hematology and clinical biochemistry assays, including blood count, glucose, HbA1c, total cholesterol, HDL-cholesterol (HDL-C), triglycerides, glutamic oxaloacetic transaminase (GOT), glutamic pyruvic transaminase (GPT), gamma-glutamyl transpeptidase (GGT), creatinine and urea were assessed by routine laboratory techniques. The Friedewald equation was used to assess LDL-cholesterol (LDL-C).

Stool samples from the various groups of patients and healthy controls were collected in tubes filled with a DNA stabilizer using the “PSP Spin Stool DNA Plus Kit” by Stratec Molecular (Berlin, Germany). A small aliquot of the sample was used for DNA extraction, as previously described [19]. The kit employs optimized buffers to efficiently remove all contaminants. The 16S sequencing was performed by an external company (IGA Technology Services Srl, Udine, Italy).

### 2.3. Sequencing and Bioinformatics Analysis of 16S Ribosomal RNA

To characterize the bacterial populations present in the samples, the V3 and V4 regions of the 16S ribosomal RNA (rRNA) gene were sequenced. The V3 and V4 regions are hypervariable regions of the 16S rRNA gene found in all bacteria; they are species-specific and thus provide phylogenetic information. The protocol for amplification and sequencing of these regions includes the following steps: (A) Amplification of the V3–V4 variable regions of the 16S rRNA (amplicons) using HIFI HotStart TAQ polymerase. (B) Purification of amplicons using magnetic beads. (C) Indexing of amplicons with Illumina INDEX tags through 8 cycles of PCR, followed by purification and quantification of indexed products. (D). Sequencing on the Illumina MiSeq platform, which reads both ends of the fragment with a central overlap of approximately 50 bp, enabling optimal reconstruction of the entire fragment.

The analysis of 16S rRNA amplicon data involves three main phases: (a) Pre-processing of sequences (reads). (b) Identification of microorganisms present in the samples. (c) Statistical analysis.

The pre-processing phase eliminates low-quality reads caused by sequencing errors. The quality of raw sequences was assessed using FastQC (version 0.12.0). Primer sequences and adapters were removed using Cutadapt (version 5.0). Preprocessed reads were analyzed using the QIIME 2 pipeline (distribution 2024.10) [20]. Specifically, reads were screened for chimeric sequences and clustered into amplicon sequence variants (ASVs) using the DADA2 (Version 1.28) algorithm [21]. Taxonomic classification of representative sequences generated by DADA2 was performed using the q2-feature-classifier plugin and the Silva database (version 138) [20,21,22].

### 2.4. Statistical Analysis

For descriptive statistical analysis, categorical variables were expressed as frequencies and percentage, and chi-squared and Fisher’s exact test with Bonferroni correction were used to assess the significance of difference between groups; continuous variables were presented as mean ± standard deviation and an independent-samples Kruskal–Wallis test was applied to assess differences between groups with Bonferroni correction for multiple testing. In all statistical evaluations, a *p* value < 0.05 was considered as significant. Statistical analyses were conducted using the SPSS Version 27.0 statistic software package for Windows (IBM SPSS Statistics for Windows, Version 23.0. IBM Corp, Armonk, NY, USA ).

Data manipulations and statistical analyses of 16S sequencing data were conducted in the R environment (version 3.6), utilizing the vegan (version 2.5.6) (https://cran.r-project.org/package=vegan, accessed on 4 September 2025) and phyloseq (version 1.30.0) (https://www.bioconductor.org/packages/release/bioc/html/phyloseq.html, accessed on 4 September 2025) packages.

α-diversity was assessed using the Chao1, Shannon, and Simpson indices to evaluate microbial richness and evenness across comparison groups. The Observed and Chao1 indices primarily measure species richness, reflecting the number of different species present in a sample. The Shannon index accounts for both species richness and evenness, providing insight into how evenly species are distributed within the community. The Inverse Simpson index also incorporates both richness and evenness but places greater emphasis on dominant species, giving more weight to the abundance of the most common taxa in a sample. Diversity estimates were calculated using the estimate_richness function from the phyloseq package, and the diversity function from the vegan package in R. Differences in α-diversity among groups was evaluated using the Kruskal–Wallis test. Post hoc pairwise comparisons were conducted using the Wilcoxon rank-sum test. To account for multiple comparisons, *p*-values from the Wilcoxon tests were adjusted using the Bonferroni correction. Results were visualized using boxplots generated with ggplot2, displaying median diversity values, interquartile ranges, and significance levels.

β-diversity was assessed using principal coordinates analysis (PCoA) based on Canberra, weighted UniFrac, unweighted UniFrac, and Bray–Curtis distance metrics. Statistical significance was evaluated using permutational multivariate analysis of variance (PERMANOVA) with 999 permutations, using the adonis2 function from the vegan package, with age and sex included as additional terms in the model to account for potential confounding. Pairwise comparisons between groups were conducted using pairwise PERMANOVA with Bonferroni correction for multiple testing. Effect sizes (R^2^) were reported to quantify the proportion of variance explained by group differences, as we have performed in previous works [22,23].

Differential abundance analysis was performed using ANCOM-BC2 Version 2.10.1 (https://www.bioconductor.org/packages/release/bioc/vignettes/ANCOMBC/inst/doc/ANCOMBC2.html, accessed on 4 September 2025) to identify microbial taxa associated with the LD phenotype while adjusting for age and sex as covariates. The analysis was conducted at the Phylum, family, and genus taxonomic levels to capture taxonomic differences across comparison groups. To adjust for compositional differences across samples, ASV counts were normalized using ANCOM-BC, which accounts for compositional bias and variance in microbiome datasets. ANCOM-BC2 was run with default normalization, using a prevalence cutoff of 10% (prv_cut = 0.1) and a library size threshold of 1000 (lib_cut = 1000) to remove low-prevalence taxa. Sensitivity analysis for zero handling was enabled (pseudo_sens = TRUE), and variance stabilization (s0_perc = 0.05) was applied. The *p*-value was adjusted for multiple comparisons using the Holm correction.

## 3. Results

### 3.1. Study Population

Participants’ characteristics are presented in Table 1. Among LD patients, all but one were affected by congenital forms (specific subtypes are listed in Supplementary Table S1), 3 (37.5%) were males and the mean age was 28.8 ± 15.4 years. Four (25%) of Ob patients were males with a mean age of 42.1 ± 10.7 years. On the basis of their metabolic syndrome status, Ob patients were further divided into MHO and MUHO subgroups.

As shown in Table 1, LD and age-matched N were younger than Ob patients and presented significantly lower BMI and waist circumference than Ob. The overall metabolic profile of LD patients appeared worse than those of N and MHO but comparable to MUHO individuals, with special regard to HDL-C, triglycerides and liver enzymes concentrations (Table 1).

Both MHO and MUHO had higher blood pressure levels than N, with MUHO also showing a worse lipid profile and more elevated liver enzyme concentration. When compared to each other, MHO and MUHO did not significantly differ in BMI, waist circumference and blood pressure, while liver enzyme levels were higher in MUHO.

### 3.2. Gut Microbiota

**Figure 1:**
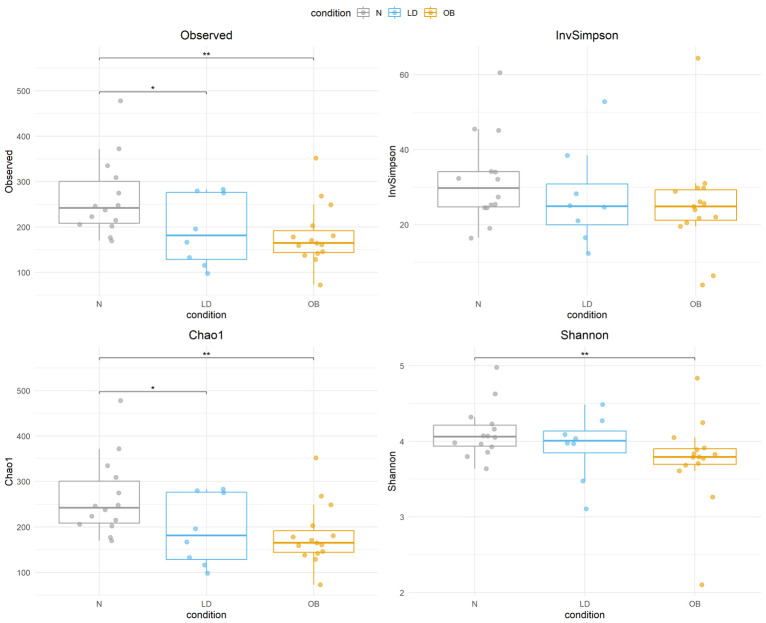
Boxplots display α-diversity indices (Observed, Inverse Simpson, Chao1, and Shannon) across N, LD, Ob groups. Each box represents the interquartile range (IQR), with the horizontal line indicating the median. Whiskers extend to 1.5 times the IQR, and individual points represent sample values. Statistical significance is denoted by asterisks (* *p* < 0.05, ** *p* < 0.01 ). Condition groups: N (normal weight controls) in gray, LD (individuals with lipodystrophy) in blue, and OB (individuals with obesity) in orange.

In this study, 41 fecal samples were analyzed, including 16 from healthy controls (N), 8 from LD patients, and 15 from Ob patients, of whom 7 were MHO and 8 were MUHO. A total of 2476 amplicon sequence variants (ASVs) were identified across all samples. The analysis was conducted in two stages: first, comparing LD individuals against the N and the Ob groups; second, stratifying the Ob group based on metabolic status into MHO and MUHO subgroups. Analysis of α-diversity revealed significantly lower diversity in the LD group compared to the N group, based on the Observed and Chao1 indices (*p* = 0.047), while no significant differences were found using the Shannon and Inverse Simpson indices (Figure 1). A significant reduction in α-diversity was also observed in the Ob group compared to the N group across all four indices (Observed *p* = 0.008, Chao1 *p* = 0.008, Shannon *p* = 0.024, Inverse Simpson *p* = 0.035). Upon stratification of the Ob group, the MUHO subgroup showed significantly lower α-diversity compared to the N group based on the Observed (*p* = 0.035) and Chao1 (*p* = 0.035) indices, but not with the Shannon or Inverse Simpson indices (Supplementary Figure S1). The MHO group showed significantly lower α-diversity compared to the N group using the Observed (*p* = 0.006), Chao1 (*p* = 0.006), and Shannon (*p* = 0.02) indices, but not the Inverse Simpson index. No significant differences were observed in the comparison between LD and Ob groups or LD and MHO or MUHO subgroups. These results indicate that α-diversity is reduced in individuals with lipodystrophy and obesity compared to healthy controls, in terms of microbial richness, as reflected by the difference in the Observed and Chao1 indices. In consideration of its different etiology, the analyses were repeated after excluding the subject with acquired generalized lipodystrophy (as reported in Supplementary Table S1), yielding consistent results .

**Figure 2:**
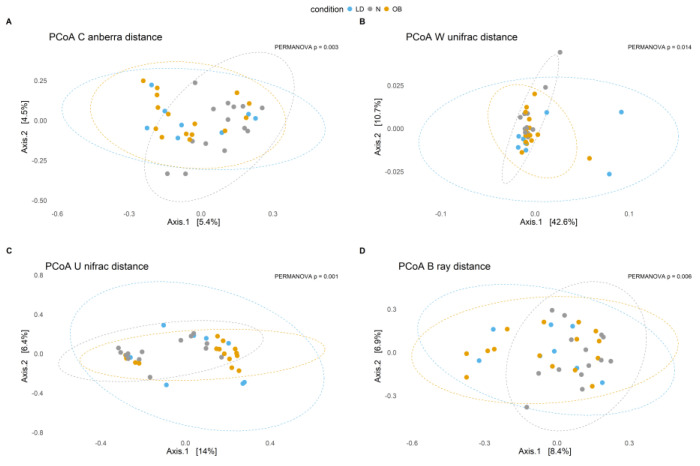
Principal Coordinates Analysis (PCoA) plots illustrate microbial community composition based on different β-diversity distance metrics across conditions. Each point represents a sample, and ellipses indicate the 95% confidence interval for each condition. The percentage on each axis represents the proportion of variance explained. Statistical significance (*p*-values) indicates differences between groups. (**A**) PCoA based on Canberra distance (*p* = 0.0026). (**B**) PCoA based on weighted UniFrac distance (*p* = 0.0107). (**C**) PCoA based on Unweighted UniFrac distance (*p* = 0.0011). (**D**) PCoA based on Bray–Curtis distance (*p* = 0.0036). Condition groups: N (normal weight controls) in gray, LD (individuals with lipodystrophy) in blue, and OB (individuals with obesity) in orange.

The β-diversity analysis indicates that the microbial composition in lipodystrophy does not markedly deviate from that of N or Ob individuals (Figure 2). The lack of significant difference between the LD and the stratified MUHO and MHO groups is also observed (Supplementary Figure S1).

Furthermore, analysis of the Firmicutes/Bacteroidetes ratio does not highlight any significant difference among all groups either considering the Ob individuals as a single group or stratifying them as MUHO and MHO groups (Supplementary Figure S3). However, a differential abundance analysis identifies interesting differences in microbial composition at different levels (Table 2).

The groups include N for normal weight, Ob for individuals with obesity, MHO for individuals with metabolically healthy obesity, MUHO for individuals with metabolically unhealthy obesity, and LD for individuals with lipodystrophy. Arrows indicate the direction of change; ↑ means increased abundance; ↓ means decreased abundance;—means no significant change.

At the phylum level, in the LD vs. N comparison, *Synergistota* was significantly increased in LD individuals (*p* < 0.05). In the LD vs. Ob comparison, *Synergistota*, *Verrucomicrobiota* and *Euryarchaeota* exhibited a significant increase (*p* < 0.05). When stratifying the Ob group, we observed that *Verrucomicrobiota* was significantly increased in LD patients in comparison to MHO (*p* < 0.05) but not in comparison to MUHO individuals.

At the family level, a significant decrease in *Synergistaceae* (phylum *Synergistota*) was observed in LD compared to Ob patients (*p* < 0.05). When further stratifying Ob individuals, we observed a significant decrease in *Burkholderiaceae* (phylum *Pseudomonadota*) in LD individuals compared to MUHO patients (*p* < 0.05).

At the genus level, a significant increase in *Parasutterella* was observed in the LD when compared with N controls, while a significant decrease in *Cloacibacillus* (phylum *Synergistota*, family *Synergistaceae*) was detected in LD individuals compared to MUHO patients (*p* < 0.05).

## 4. Discussion

In this study, we compared LD with Ob patients (further classified as MHO or MUHO based on metabolic status) and normal-weight controls. Our hypothesis was that, since LD patients share similar metabolic impairments with Ob patients, they might also exhibit comparable gut microbiota dysbiosis.

From a metabolic perspective, our findings confirm that, despite their distinct phenotypes, both LD and Ob patients share common metabolic impairments. In obesity, subcutaneous adipose tissue plasticity and expandability can be limited over time by the progression of adiposopathy [24], leading to free fatty acids spillover, ectopic fat deposition and obesity-related metabolic complication development. This progression sets the pace of the clinical transition from MHO to MUHO phenotypes [25]. Similarly, in LD, the absence or reduction in subcutaneous adipose tissue forces the storage of surplus energy, further exacerbated by hyperphagia due to low leptin levels [26], into visceral and other ectopic sites, triggering lipotoxicity and insulin resistance [27]. In the liver, hyperinsulinemia promotes de novo lipogenesis, inflammation, and cellular senescence, leading to metabolic-associated steatotic liver disease (MASLD), which has now outreached alcohol-related and viral liver diseases as the leading cause of chronic liver disease [28]. Thus, liver damage may serve as a warning sign of metabolic impairment in both LD and Ob patients. Accordingly, in our cohort, we found elevated liver enzymes levels in both LD and MUHO individuals but not in MHO. The metabolic impairment observed in LD patients was more pronounced than in N and MHO individuals, but comparable to that of MUHO individuals, also in terms of HDL-C concentrations.

A growing body of evidence suggests that intestinal dysbiosis is associated with metabolic diseases, even though the causal relationship between them has not been fully elucidated. Dysbiosis consists of the loss of overall microbiota diversity and alteration in its composition, promoting host energy homeostatic changes, low-grade inflammation [10], and impairment of protection against harmful microorganisms [29], which may culminate in predisposition to the development of insulin resistance and obesity [30], as demonstrated by several microbiota transplantation experiments [31,32]. Importantly, gut microbiota composition is strongly shaped by external factors, particularly diet and medication use. Diets high in saturated fats and refined carbohydrates and low in fiber are associated with reduced microbial diversity and pro-inflammatory profiles, whereas fiber-rich diets favor taxa linked to metabolic health. Likewise, not only commonly prescribed medications, including antibiotics, metformin, and proton pump inhibitors, but also food additives, such as artificial sweeteners, can alter gut microbial composition [33]. These factors may act as important modifiers or confounders in microbiota–metabolism associations and should be considered when interpreting microbiome data. At the same time, this sensitivity to dietary and pharmacological exposures underscores the gut microbiota as a modifiable therapeutic target, with microbiome-directed interventions representing promising strategies to improve metabolic and inflammatory outcomes [34].

In this study, gut microbiota analysis indicates that α-diversity is reduced in individuals with lipodystrophy and obesity compared to healthy controls. More specifically, α-diversity is significantly lower in LD compared to the N group, based on the Observed and Chao1 indices (*p* = 0.047), but not on the Shannon and Inverse Simpson indices. A similar α-diversity trend is observed when comparing the MUHO and N individuals, whilst the MHO subgroup showed significantly lower α-diversity compared to the N for the Observed (*p* = 0.006), Chao1 (*p* = 0.006), and Shannon (*p* = 0.02) indices, but not for the Inverse Simpson index. The lack of significant differences in the Inverse Simpson index, a metric more sensitive to dominant taxa than to overall richness, indicates that dominant taxa remain relatively stable across conditions. It is likely that differences in microbial composition may be primarily driven by shifts in rare taxa rather than changes in the most abundant species. This result is confirmed by the Shannon index, that is weakly significant only for the MHO and N comparison, indicating that microbial evenness remains relatively stable, even as species richness declines, suggesting a variation in rare taxa while the relative abundance of the dominant species remains stable. Overall, these findings highlight metabolic health status as a key determinant of microbial richness, while microbial evenness remains relatively unaffected. The reduction in α-diversity observed primarily in richness-based metrics (Observed and Chao1), together with the limited changes in Shannon and Inverse Simpson indices, suggests that the overall structure of dominant taxa may remain relatively preserved, whereas less abundant members of the community could be preferentially depleted. This pattern is consistent with a loss of microbial “reservoir” diversity that may reduce ecosystem resilience and functional redundancy, which are features that have been linked to metabolic dysfunction and low-grade inflammation. Notably, α-diversity comparisons were performed using non-parametric tests (Kruskal–Wallis with post hoc Wilcoxon tests), which do not allow adjustment for age and sex; therefore, residual confounding by age cannot be completely excluded when interpreting α-diversity results.

The β-diversity analysis shows an absence of significant differences between LD and either N or Ob group. This finding suggests that variation in microbial richness, highlighted by difference in α-diversity, may not necessarily translate into significant compositional differences between groups. Thus, β-diversity metrics, particularly UniFrac and Bray–Curtis distances, are mainly influenced by the most abundant taxa. This comparison was evaluated using PERMANOVA including age and sex as covariates, thereby reducing potential confounding due to the older age of the obesity groups.

It is worth mentioning that, among the investigated groups, there is not a significant difference in the Firmicutes/Bacteroidetes ratio. It is likely that compositional changes at genus or species level may be more relevant than the Firmicutes/Bacteroidetes ratio for association with a given health condition [13]. In any case, our data indicates that the Firmicutes/Bacteroidetes ratio cannot be used as a microbial biomarker to distinguish metabolically unhealthy from healthy individuals. In line with our data, in the only previous study that evaluated gut microbiota diversity in 17 patients with congenital generalized lipodystrophy, α-diversity was found reduced compared to controls, while no significant differences were observed in β-diversity or in the Firmicutes/Bacteroidetes ratio [17].

The differential abundant analysis revealed significant variations in microbial composition across LD, N, and Ob individuals. A notable observation was the increased abundance of *Synergistota* in LD individuals compared to N and Ob groups. This phylum has been linked to inflammatory conditions, and its presence in LD individuals may reflect microbiome adaptations to metabolic disturbances associated with lipodystrophy, which is characterized by severe insulin resistance, ectopic fat accumulation, and systemic inflammation. When further stratifying Ob individuals into MHO and MUHO groups, *Synergistota* remained significantly higher in LD compared to MHO and similar to MUHO individuals. This suggests that *Synergistota* is not merely associated with obesity but may be more closely linked to metabolic dysfunction and inflammatory processes. The similarity in *Synergistota* abundance between LD and MUHO individuals reinforces the idea that this phylum may thrive in environments characterized by systemic metabolic disturbances. Supporting this notion, previous research has reported an increased presence of *Synergistota* in individuals with diabetic retinopathy, which is a condition closely linked to insulin resistance and systemic inflammation [35].

Another key finding was the increased presence of *Verrucomicrobiota* in LD individuals compared to Ob individuals, and, in particular, to the MHO subgroup. This phylum is largely driven by *Akkermansia muciniphila*, a mucus-associated commensal that has been linked to improved gut barrier integrity and metabolic/inflammatory regulation [36]. Consistently, *A*. *muciniphila* is reported to be reduced in uncomplicated obesity [37,38], and the reduced abundance of *Verrucomicrobiota* in Ob (including both MHO and MUHO) may indicate a loss of potentially protective microbial functions [39,40]. However, in states of severe metabolic derangement and dysbiosis (e.g., LD and complicated obesity), an increased representation of mucus-degrading taxa may also reflect barrier stress; in particular, low-fiber–associated mucus erosion has been linked to thinning of the mucus layer and increased epithelial vulnerability [41], and experimental over-colonization of *A*. *muciniphila* has been shown to reduce mucus thickness and impair tight junction/barrier markers [42,43]. Because mucus fermentation contributes to short-chain fatty acid (SCFA) production [44], and microbiota-derived acetate can fuel hepatic de novo lipo-genesis in specific dietary/metabolic contexts [45], the enrichment of mucus-degrading taxa may have adverse downstream metabolic consequences in dysbiotic states. Thus, the relative enrichment of *Verrucomicrobiota* observed in LD should be interpreted cautiously as a potential maladaptive dysbiotic feature rather than unequivocally protective [43]. The concurrent reduction in *Euryarchaeota*, which includes methanogenic archaea such as *Methanobrevibacter*, in Ob individuals compared to LD suggests potential alterations in microbial fermentation and energy extraction that may influence obesity-related metabolic changes.

The increased abundance of *Synergistaceae*, a family within *Synergistota*, in Ob individuals compared to LD suggests that the gut microbiome may undergo adaptive changes in response to the altered metabolic environment of obesity. The increased presence of *Burkholderiaceae* in MUHO individuals, compared to LD, highlights a potential microbial signature associated with metabolically unhealthy obesity. *Burkholderiaceae*, which belongs to *Pseudomonadota* (formerly *Proteobacteria*), has been associated with systemic inflammation and metabolic disorders [46,47].

At the genus level, the increased abundance of *Parasutterella* in LD individuals compared to N suggests a potential microbial signature linked to bile acid metabolism and gut homeostasis, which are processes that could influence lipid and glucose homeostasis in lipodystrophy [48,49]. Conversely, the increased presence of *Cloacibacillus* in MUHO individuals compared to LD may indicate a shift toward a more inflammatory microbial profile. *Cloacibacillus*, an anaerobic bacterium from the *Synergistaceae* family, has been associated with inflammatory and dysbiotic gut environments, supporting the idea that metabolically unhealthy obesity is characterized by a microbiome profile that may exacerbate metabolic dysfunction.

Taken together, these findings suggest that LD individuals have a distinct gut microbiome signature that differentiates them from both normal-weight persons and individuals with obesity, with microbial alterations that may be linked to metabolic dysfunction rather than obesity per se. While our results provide further evidence of an association between gut microbiota alterations and metabolic impairment, several aspects warrant further investigation. Future research should integrate multi-omics approaches, including metabolomics, to characterize host–microbe metabolic interactions, and employ functional profiling via metagenomics and metatranscriptomics to move beyond taxonomic associations, identifying pathways relevant to metabolic and inflammatory regulation [50]. The gut microbiota’s responsiveness to environmental factors also highlights its potential as a therapeutic target [34]. Microbiome-directed interventions, such as dietary modulation, prebiotics or probiotics, may improve metabolic health also in congenital diseases like LD, but longitudinal and interventional studies are needed to establish causality, identify responders, and confirm long-term efficacy and safety of such interventions.

Finally, some limitations of this study should be acknowledged. First, the small sample size of participants with LD reflecting the rarity of the disease limits statistical power. In addition, the inclusion of different LD variants precluded subtype-specific analyses. Larger cohorts will be required to determine whether distinct LD subtypes exhibit unique metabolic or gut microbiota profiles. Increasing the sample size would also enable a more robust comparison of gut microbiota similarity between LD and MUHO versus MHO. Second, although the gut microbiota is relatively stable during adulthood after early-life establishment and is largely influenced by environmental factors such as diet, metabolic status, lifestyle, and by comorbidities [51], age-related effects cannot be fully excluded. Future studies employing age-matched cohorts and longitudinal design will be necessary to disentangle the independent contribution of age to gut microbiota alterations. Finally, the cross-sectional design limits causal inference; therefore, it is not possible to determine whether differences in metabolic status are a consequence of gut microbiota composition or vice versa.

## 5. Conclusions

In our study, we analyzed metabolic aspects and gut microbiota composition in individuals with lipodystrophy, comparing them to individuals with obesity and normal-weight controls. Our findings confirm that, despite their distinct phenotypic characteristics, individuals with lipodystrophy exhibit metabolic impairments similar to those observed in MUHO individuals.

Gut microbiota analysis revealed a reduction in microbial diversity (α-diversity2 in both LD and Ob compared to normal-weight controls but absence of significant differences in β-diversity between LD and either N or Ob group, suggesting that metabolic dysfunction is more linked to the loss of microbial diversity rather than to specific compositional changes. Moreover, the Firmicutes/Bacteroidetes ratio, often considered a metabolic health marker, did not prove to be a reliable discriminator in our study population.

In conclusion, the relationship between gut microbiota and metabolism is complex and multifactorial and cannot be reduced to a single ratio or parameter. The role of specific taxa, such as *Verrucomicrobiota* (particularly *Akkermansia muciniphila*) warrants further investigation to assess their potential impact on metabolic health. Beyond microbial composition, it is essential to explore the functional profile of the microbiota by analyzing microbial-derived metabolites (e.g., short chain fatty acids and lipopolysaccharide) and their effects on inflammation, lipid metabolism, and insulin sensitivity to better understand the development of adiposopathy both in lipodystrophy and obesity. Larger studies would be needed to allow for a more precise characterization of different lipodystrophy subtypes.

## Figures and Tables

**Table 1:** Patients’ characteristics and comparisons between groups.

	LD (*n* = 8)	N (*n* = 16)	Ob (*n* = 16)	MHO (*n* = 8)	MUHO (*n* = 8)	LD vs. N	LD vs. Ob	N vs. Ob	LD vs. MHO	LD vs. MUHO	N vs. MHO	N vs. MUHO	MHO vs. MUHO
**Males (%)**	37.5%	25%	25%	0%	50%	ns	ns	ns	ns	ns	ns	ns	<0.05
**Age (years)**	28.8 (15.4)	30.9 (4.7)	42.1 (10.7)	41.5 (10.0)	42.6 (12.1)	ns	<0.05	<0.01	<0.05	<0.05	<0.05	<0.05	ns
**BMI (kg/m^2^)**	16.8 (4.6)	21.0 (2.0)	37.3 (5.7)	35.5 (4.9)	39.2 (6.2)	ns	<0.001	<0.001	<0.001	<0.001	0.001	<0.001	ns
**Waist circumference (cm)**	65.3 (19.0)	76 (5.4)	113.5 (14.7)	106.6 (5.8)	122.7 (18.3)	ns	<0.001	<0.001	<0.001	<0.001	0.001	<0.001	ns
**SBP (mmHg)**	111.6 (13.9)	108.1 (8.5)	123.1 (11.5)	120.6 (10.8)	125.6 (12.4)	ns	<0.05	<0.001	ns	<0.05	<0.05	<0.01	ns
**DBP (mmHg)**	72.3 (14.0)	61.9 (6.3)	76.3 (9.2)	73.8 (8.8)	78.8 (9.5)	0.07	ns	<0.001	ns	ns	<0.01	<0.001	ns
**Heart rate (bpm)**	73.0 (14.0)	69.6 (6.7)	74.2 (9.2)	72 (4.7)	76.4 (12.1)	ns	ns	ns	ns	ns	ns	ns	ns
**Antipertensive treatment**	25%	0%	56.3%	37.5%	75%	ns	ns	<0.001	ns	0.07	<0.05	<0.001	ns
**Dyslipidemia treatment**	50%	0%	12.5%	12.5%	12.5%	<0.05	ns	ns	ns	ns	ns	ns	ns
**Fasting glucose (mg/dL)**	87.5 (15.3)	86.4 (7.3)	94.7 (13.5)	88.1 (8.7)	100.5 (14.7)	ns	ns	ns	<0.01	0.07	ns	<0.05	0.08
**Hb1Ac (mmol)**	44.6 (25.7)	31.5 (3.5)	35.2 (4.0)	34.1 (2.7)	36.5 (5.0)	ns	ns	ns	ns	ns	ns	ns	ns
**Total cholesterol (mg/dL)**	164.8 (25.9)	165.1 (30.3)	195.1 (53.0)	180.6 (27.6)	207.8 (67.6)	ns	ns	ns	ns	ns	ns	ns	ns
**HDL-C (mg/dL)**	42.3 (15.6)	63 (9.3)	51.2 (13.8)	58.6 (13.7)	44.8 (10.7)	<0.001	ns	<0.05	<0.05	ns	ns	<0.01	0.08
**LDL-C (mg/dL)**	102.2 (22.7)	89.9 (26.5)	128.5 (40.5)	109.4 (23.0)	145.1 (46.3)	ns	ns	<0.01	ns	0.07	0.10	<0.001	ns
**Triglycerides (mg/dL)**	138.8 (131)	61.1 (35.2)	105.7 (84.9)	65.9 (26.7)	140.6 (104.1)	<0.01	ns	<0.05	ns	ns	ns	<0.01	0.09
**GPT (U/L)**	42.9 (49.9)	19.7 (7.1)	39.9 (33.3)	21.1 (10.8)	56.4 (38.2)	ns	ns	ns	ns	ns	ns	<0.01	<0.01
**GOT (U/L)**	46.1 (37.5)	22.3 (5)	32.9 (23.2)	22.7 (17.9)	41.9 (22.9)	ns	ns	ns	<0.01	ns	ns	<0.01	<0.01
**GGT (U/L)**	41.8 (43.0)	17.1 (5.8)	46 (44.8)	20.6 (11.5)	109.5 (43.1)	ns	ns	ns	ns	ns	ns	<0.05	<0.05

Abbreviations: DBP, diastolic blood pressure; SBP, systolic blood pressure. Data are presented as mean (standard deviation) or percentages. An independent-samples Kruskal–Wallis test was applied for pairwise comparisons between groups with Bonferroni correction for multiple testing. Pairwise comparisons of categorical variables across groups were performed using Fisher’s exact test with Bonferroni correction. *p* values < 0.10 were reported. ns, not significant.

**Table 2:** Microbial taxa with significant abundance changes across different comparisons. ↑, increase; ↓, decrease.

Level	Taxa	LD vs. N	LD vs. Ob	LD vs. MHO	LD vs. MUHO
Phylum	*Synergistota*	↑	↑	–	–
Phylum	*Verrucomicrobiota*	–	↑	↑	–
Phylum	*Euryarcheota*	–	↑	–	–
Family	*Synergistaceae*	–	↓	–	–
Family	*Burkholderiaceae*	–	–	–	↑
Genus	*Parasutterella*	↑	–	–	–
Genus	*Cloacibacillus*	–	–	–	↑

## Data Availability

The raw data supporting the conclusions of this article will be made available by the authors on request.

## Supplementary Materials

The following supporting information can be downloaded at: https://www.mdpi.com/article/10.3390/microorganisms14010132/s1; Figure S1: α-diversity across N, LD, MHO, and MUHO metabolic states; Figure S2: β-diversity across N, LD, MHO and MUHO metabolic states; Figure S3: Firmicutes/Bacteroidetes ratio across N, LD, MHO and MUHO metabolic states; Table S1: Demographic data and lipodystrophy subtypes.

## References

[1] Akinci B., von Schnurbein J., Araujo-Vilar D., Wabitsch M., Oral E.A. (2024). Lipodystrophy Prevalence, “Lipodystrophy-Like Phenotypes,” and Diagnostic Challenges. Diabetes.

[2] Ceccarini G., Magno S., Gilio D., Pelosini C., Santini F. (2021). Autoimmunity in lipodystrophy syndromes. Presse Med..

[3] Tews D., Schulz A., Denzer C., von Schnurbein J., Ceccarini G., Debatin K.M., Wabitsch M. (2021). Lipodystrophy as a Late Effect after Stem Cell Transplantation. J. Clin. Med..

[4] Lim K., Haider A., Adams C., Sleigh A., Savage D.B. (2021). Lipodistrophy: A paradigm for understanding the consequences of “overloading” adipose tissue. Physiol. Rev..

[5] Sbraccia P., D’Adamo M., Guglielmi V. (2021). Is type 2 diabetes an adiposity-based metabolic disease? From the origin of insulin resistance to the concept of dysfunctional adipose tissue. Eat. Weight. Disord..

[6] Tanriover C., Copur S., Gaipov A., Ozlusen B., Akcan R.E., Kuwabara M., Hornum M., Van Raalte D.H., Kanbay M. (2023). Metabolically healthy obesity: Misleading phrase or healthy phenotype?. Eur. J. Intern. Med..

[7] Eckel N., Li Y., Kuxhaus O., Stefan N., Hu F.B., Schulze M.B. (2018). Transition from metabolic healthy to unhealthy phenotypes and association with cardiovascular disease risk across BMI categories in 90 257 women (the Nurses’ Health Study): 30 year follow-up from a prospective cohort study. Lancet Diabetes Endocrinol..

[8] Zhao J.Y., Zhou L.J., Ma K.L., Hao R., Li M. (2024). MHO or MUO? White adipose tissue remodeling. Obes. Rev..

[9] Le Chatelier E., Nielsen T., Qin J., Prifti E., Hildebrand F., Falony G., Almeida M., Arumugam M., Batto J.M., Kennedy S. (2013). Richness of human gut microbiome correlates with metabolic markers. Nature.

[10] Colangeli L., Escobar Marcillo D.I., Simonelli V., Iorio E., Rinaldi T., Sbraccia P., Fortini P., Guglielmi V. (2023). The Crosstalk between Gut Microbiota and White Adipose Tissue Mitochondria in Obesity. Nutrients.

[11] Ramadan Y.N., Alqifari S.F., Alshehri K., Alhowiti A., Mirghani H., Alrasheed T., Aljohani F., Alghamdi A., Hetta H.F. (2025). Microbiome Gut-Brain-Axis: Impact on Brain Development and Mental Health. Mol. Neurobiol..

[12] Hetta H.F., Ramadan Y.N., Alharbi A.A., Alsharef S., Alkindy T.T., Alkhamali A., Albalawi A.S., El Amin H. (2024). Gut Microbiome as a Target of Intervention in Inflammatory Bowel Disease Pathogenesis and Therapy. Immuno.

[13] Magne F., Gotteland M., Gauthier L., Zazueta A., Pesoa S., Navarrete P., Balamurugan R. (2020). The Firmicutes/Bacteroidetes Ratio: A Relevant Marker of Gut Dysbiosis in Obese Patients?. Nutrients.

[14] Kim M.H., Yun K.E., Kim J., Park E., Chang Y., Ryu S., Kim H.L., Kim H.N. (2020). Gut microbiota and metabolic health among overweight and obese individuals. Sci. Rep..

[15] Aljuraiban G.S., Alfhili M.A., Aldhwayan M.M., Aljazairy E.A., Al-Musharaf S. (2023). Shared and Distinct Gut Microbial Profiles in Saudi Women with Metabolically Healthy and Unhealthy Obesity. Microorganisms.

[16] Alcazar M., Escribano J., Ferré N., Closa-Monasterolo R., Selma-Royo M., Feliu A., Castillejo G., Luque V., Feliu-Rovira A., Muñoz-Hernando J. (2022). Gut microbiota is associated with metabolic health in children with obesity. Clin. Nutr..

[17] Montenegro Junior R.M., Ponte C.M.M., Castelo M., de Oliveira Silveira A.C., Fernandes V.O., D’Alva C.B., Oliveira L.F.V., Hristov A.D., Bandeira S.P., da Cruz Paiva G.E. (2022). Reduced gut microbiota diversity in patients with congenital generalized lipodystrophy. Diabetol. Metab. Syndr..

[18] Grundy S.M., Becker D., Clark L.T., Cooper R.S., Denke M.A., Howard W.J., Hunninghake D.B., Illingworth R., Luepker R.V., McBride P. (2002). Third Report of the National Cholesterol Education Program (NCEP) Expert Panel on Detection, Evaluation, and Treatment of High Blood Cholesterol in Adults (Adult Treatment Panel III) final report. Circulation.

[19] Pietrucci D., Teofani A., Unida V., Cerroni R., Biocca S., Stefani A., Desideri A. (2020). Can Gut Microbiota Be a Good Predictor for Parkinson’s Disease? A Machine Learning Approach. Brain Sci..

[20] Bolyen E., Rideout J.R., Dillon M.R., Bokulich N.A., Abnet C.C., Al-Ghalith G.A., Alexander H., Alm E.J., Arumugam M., Asnicar F. (2019). Reproducible, interactive, scalable and extensible microbiome data science using QIIME 2. Nat. Biotechnol..

[21] Callahan B.J., McMurdie P.J., Rosen M.J., Han A.W., Johnson A.J., Holmes S.P. (2016). DADA2: High-resolution sample inference from Illumina amplicon data. Nat. Methods.

[22] Cerroni R., Pietrucci D., Teofani A., Chillemi G., Liguori C., Pierantozzi M., Unida V., Selmani S., Mercuri N.B., Stefani A. (2022). Not just a Snapshot: An Italian Longitudinal Evaluation of Stability of Gut Microbiota Findings in Parkinson’s Disease. Brain Sci..

[23] Teofani A., Libonati A., Unida V., Biocca S., Desideri A., Campanella V. (2024). Coronal and Root Canal Microbiota in Apical Periodontitis with Different PAI. Microorganisms.

[24] Guglielmi V., Cardellini M., Cinti F., Corgosinho F., Cardolini I., D’Adamo M., Zingaretti M.C., Bellia A., Lauro D., Gentileschi P. (2015). Omental adipose tissue fibrosis and insulin resistance in severe obesity. Nutr. Diabetes.

[25] Iacobini C., Pugliese G., Blasetti Fantauzzi C., Federici M., Menini S. (2019). Metabolically healthy versus metabolically unhealthy obesity. Metabolism.

[26] O’Rahilly S. (2021). “Treasure Your Exceptions”-Studying Human Extreme Phenotypes to Illuminate Metabolic Health and Disease: The 2019 Banting Medal for Scientific Achievement Lecture. Diabetes.

[27] Ficarella R., Laviola L., Giorgino F. (2015). Lipodystrophic diabetes mellitus: A lesson for other forms of diabetes?. Curr. Diabetes Rep..

[28] Basil B., Myke-Mbata B.K., Eze O.E., Akubue A.U. (2024). From adiposity to steatosis: Metabolic dysfunction-associated steatotic liver disease, a hepatic expression of metabolic syndrome—Current insights and future directions. Clin. Diabetes Endocrinol..

[29] Petersen C., Round J.L. (2014). Defining dysbiosis and its influence on host immunity and disease. Cell. Microbiol..

[30] Breton J., Galmiche M., Déchelotte P. (2022). Dysbiotic Gut Bacteria in Obesity: An Overview of the Metabolic Mechanisms and Therapeutic Perspectives of Next-Generation Probiotics. Microorganisms.

[31] Hur K.Y., Lee M.S. (2015). Gut Microbiota and Metabolic Disorders. Diabetes Metab. J..

[32] Ridaura V.K., Faith J.J., Rey F.E., Cheng J., Duncan A.E., Kau A.L., Griffin N.W., Lombard V., Henrissat B., Bain J.R. (2013). Gut microbiota from twins discordant for obesity modulate metabolism in mice. Science.

[33] Hetta H.F., Sirag N., Elfadil H., Salama A., Aljadrawi S.F., Alfaifi A.J., Alwabisi A.N., AbuAlhasan B.M., Alanazi L.S., Aljohani Y.A. (2025). Artificial Sweeteners: A Double-Edged Sword for Gut Microbiome. Diseases.

[34] Lai S., Molfino A., Testorio M., Perrotta A.M., Currado A., Pintus G., Pietrucci D., Unida V., La Rocca D., Biocca S. (2019). Effect of Low-Protein Diet and Inulin on Microbiota and Clinical Parameters in Patients with Chronic Kidney Disease. Nutrients.

[35] Bai J., Wan Z., Zhang Y., Wang T., Xue Y., Peng Q. (2022). Composition and diversity of gut microbiota in diabetic retinopathy. Front. Microbiol..

[36] Chelakkot C., Choi Y., Kim D.K., Park H.T., Ghim J., Kwon Y., Jeon J., Kim M.S., Jee Y.K., Gho Y.S. (2018). Akkermansia muciniphila-derived extracellular vesicles influence gut permeability through the regulation of tight junctions. Exp. Mol. Med..

[37] Zhou Q., Zhang Y., Wang X., Yang R., Zhu X., Zhang Y., Chen C., Yuan H., Yang Z., Sun L. (2020). Gut bacteria Akkermansia is associated with reduced risk of obesity: Evidence from the American Gut Project. Nutr. Metab..

[38] Dao M.C., Belda E., Prifti E., Everard A., Kayser B.D., Bouillot J.L., Chevallier J.M., Pons N., Le Chatelier E., Ehrlich S.D. (2019). Akkermansia muciniphila abundance is lower in severe obesity, but its increased level after bariatric surgery is not associated with metabolic health improvement. Am. J. Physiol. Endocrinol. Metab..

[39] Yan J., Sheng L., Li H. (2021). Akkermansia muciniphila: Is it the Holy Grail for ameliorating metabolic diseases?. Gut Microbes.

[40] Segers A., de Vos W.M. (2023). Mode of action of Akkermansia muciniphila in the intestinal dialogue: Role of extracellular proteins, metabolites and cell envelope components. Microbiome Res. Rep..

[41] Desai M.S., Seekatz A.M., Koropatkin N.M., Kamada N., Hickey C.A., Wolter M., Pudlo N.A., Kitamoto S., Terrapon N., Muller A. (2016). A Dietary Fiber-Deprived Gut Microbiota Degrades the Colonic Mucus Barrier and Enhances Pathogen Susceptibility. Cell.

[42] Qu S., Zheng Y., Huang Y., Feng Y., Xu K., Zhang W., Wang Y., Nie K., Qin M. (2023). Excessive consumption of mucin by over-colonized Akkermansia muciniphila promotes intestinal barrier damage during malignant intestinal environment. Front. Microbiol..

[43] Mierlan O.L., Busila C., Amaritei O., Elena D., Raileanu C.R., Maftei N.M., Matei M.N., Gurau G. (2025). Akkermansia muciniphila in Metabolic Disease: Far from Perfect. Int. J. Mol. Sci..

[44] Morrison D.J., Preston T. (2016). Formation of short chain fatty acids by the gut microbiota and their impact on human metabolism. Gut Microbes.

[45] Zhao S., Jang C., Liu J., Uehara K., Gilbert M., Izzo L., Zeng X., Trefely S., Fernandez S., Carrer A. (2020). Dietary fructose feeds hepatic lipogenesis via microbiota-derived acetate. Nature.

[46] Wiersinga W.J., de Vos A.F., de Beer R., Wieland C.W., Roelofs J.J., Woods D.E., Van Der Poll T. (2008). Inflammation patterns induced by different Burkholderia species in mice. Cell. Microbiol..

[47] Burcelin R. (2016). Gut microbiota and immune crosstalk in metabolic disease. Mol. Metab..

[48] Henneke L., Schlicht K., Andreani N.A., Hollstein T., Demetrowitsch T., Knappe C., Hartmann K., Jensen-Kroll J., Rohmann N., Pohlschneider D. (2022). A dietary carbohydrate—Gut Parasutterella—Human fatty acid biosynthesis metabolic axis in obesity and type 2 diabetes. Gut Microbes.

[49] Ju T., Kong J.Y., Stothard P., Willing B.P. (2019). Defining the role of Parasutterella, a previously uncharacterized member of the core gut microbiota. ISME J..

[50] Tegegne H.A., Savidge T.C. (2025). Gut microbiome metagenomics in clinical practice: Bridging the gap between research and precision medicine. Gut Microbes.

[51] Schoultz I., Claesson M.J., Dominguez-Bello M.G., Fåk Hållenius F., Konturek P., Korpela K., Laursen M.F., Penders J., Roager H., Vatanen T. (2025). Gut microbiota development across the lifespan: Disease links and health-promoting interventions. J. Intern. Med..

