# Supplementary material for: Gut Microbiota in Lipodystrophies and Obesity: A Common Signature?
Source: Microorganisms. 2026 Jan 7;14(1):132. doi: 10.3390/microorganisms14010132 (PMC12843611; doi:10.3390/microorganisms14010132)
Supplement: Supplementary file 1 [file microorganisms-14-00132-s001.zip › microorganisms-3999917-supplementary.pdf]

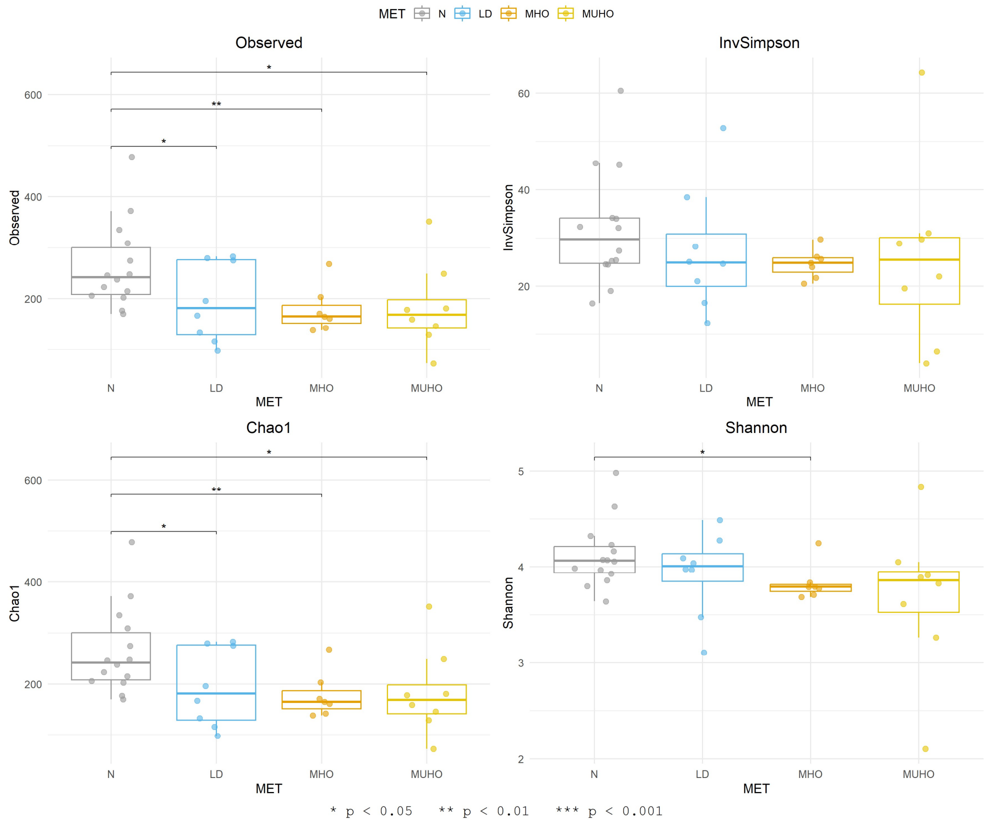

Supplementary figure S1. Boxplots display α-diversity indices (Observed, Inverse Simpson, Chao1, and Shannon) across N, LD, MHO and MUHO metabolic states. Each box represents the interquartile range (IQR), with the horizontal line indicating the median. Whiskers extend to 1.5 times the IQR, and individual points represent sample values. Statistical significance is denoted by asterisks (* $p < 0.05$, ** $p < 0.01$, *** $p < 0.001$). Condition groups: N (normal weight controls) in gray, LD (individuals with lipodystrophy) in blue, MHO (individuals with metabolically healthy obesity) in orange, MUHO (individuals with metabolically unhealthy obesity) in yellow.

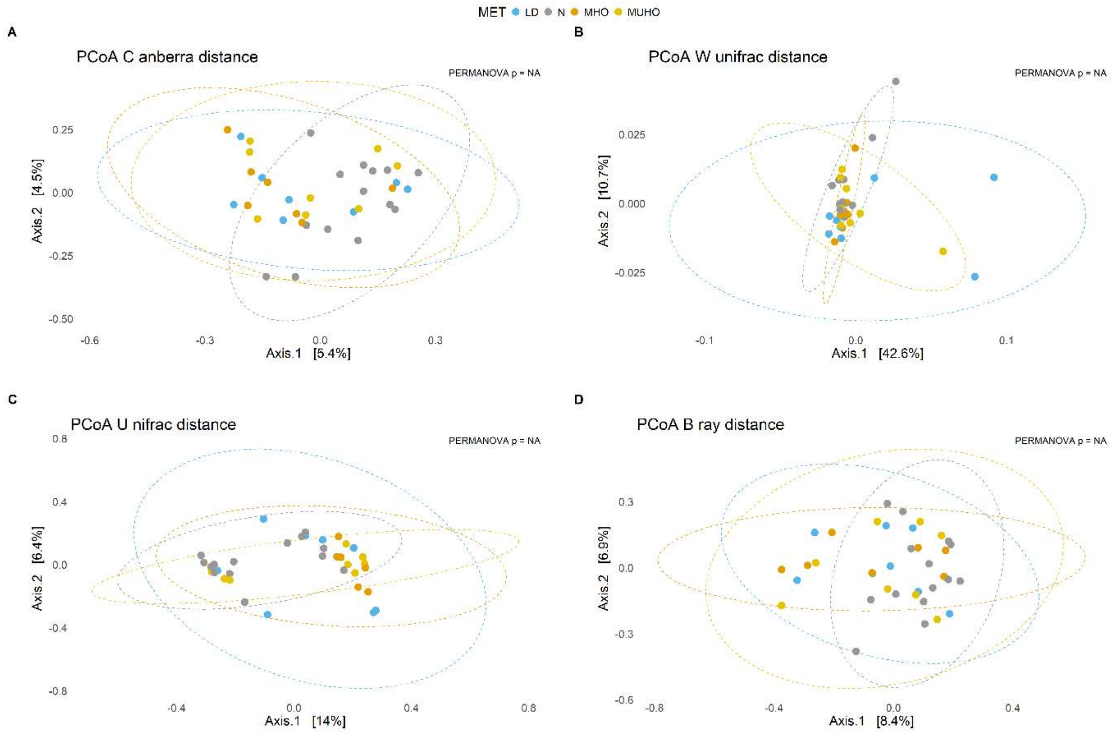

Supplementary figure S2. Principal Coordinates Analysis (PCoA) plots illustrate microbial community composition based on different beta diversity distance metrics across metabolic conditions. Each point represents a sample, and ellipses indicate the 95% confidence interval for each condition. The percentage on each axis represents the proportion of variance explained. Statistical significance was assessed using PERMANOVA (p-values reported). A) PCoA based on Canberra distance (p = 0.005). B) PCoA based on Weighted UniFrac distance (p = 0.012). C) PCoA based on Unweighted UniFrac distance (p = 0.006). D) PCoA based on Bray-Curtis distance (p = 0.009). Condition groups: N (normal weight controls) in gray, LD (individuals with lipodystrophy) in blue, MHO (individuals with metabolically healthy obesity) in orange, and MUHO (individuals with metabolically unhealthy obesity) in yellow.

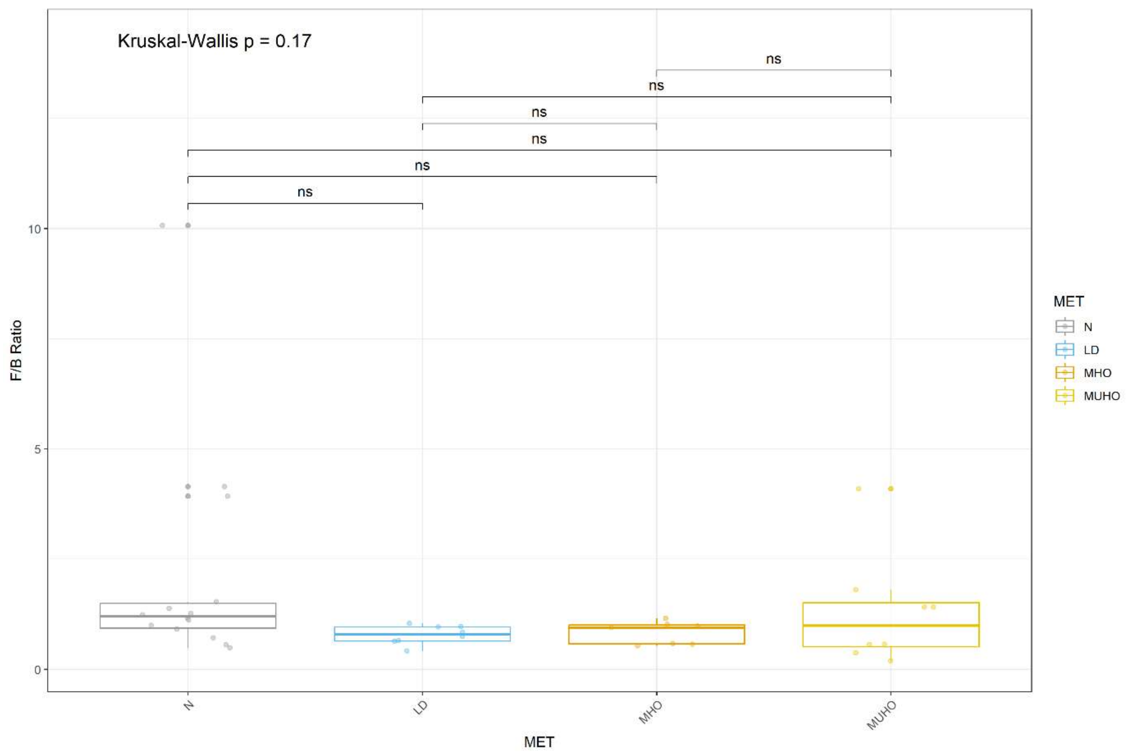

Supplementary figure S3. Boxplots display Firmicutes/Bacteroidetes ratio across N, LD, MHO and MUHO metabolic states. Each box represents the interquartile range (IQR), with the horizontal line indicating the median. Whiskers extend to 1.5 times the IQR, and individual points represent sample values. ns, non significant. Condition groups: N (normal weight controls) in gray, LD (individuals with lipodystrophy) in blue, MHO (individuals with metabolically healthy obesity) in orange, MUHO (individuals with metabolically unhealthy obesity) in yellow.

	Sex	Age	Diagnosis
**LD1**	M	22	Mandibuloacral dysplasia type A with partial lipodystrophy (MAD-A)
**LD2**	F	53	Lawrence's syndrome (acquired generalized lipodystrophy)
**LD3**	F	39	Mandibuloacral dysplasia type A with partial lipodystrophy (MAD-A)
**LD4**	F	25	Mandibuloacral dysplasia type A with partial lipodystrophy (MAD-A)
**LD5**	F	31	Mandibular hypoplasia, deafness and progeroid features with concomitant Lipodystrophy (MDPL syndrome)
**LD6**	M	11	Mandibular hypoplasia, deafness and progeroid features with concomitant Lipodystrophy (MDPL syndrome)
**LD7**	F	7	Mandibular hypoplasia, deafness and progeroid features with concomitant Lipodystrophy (MDPL syndrome)
**LD8**	M	41	Congenital generalized lipodystrophy.

Supplementary table S1. Demographic data and lipodystrophy subtypes.
